# A Polypyrrole-based Strain Sensor Dedicated to Measure Bladder Volume in Patients with Urinary Dysfunction

**DOI:** 10.3390/s8085081

**Published:** 2008-08-27

**Authors:** Sumitra Rajagopalan, Mohamad Sawan, Ebrahim Ghafar-Zadeh, Oumarou Savadogo, Vamsy P. Chodavarapu

**Affiliations:** 1 Polystim Neurotechnologies Laboratory, Department of Electrical Engineering, École Polytechnique de Montréal, Montréal, Quebec, Canada H3C 3A7; E-mails: sumitra.rajagopalan@polymtl.ca; mohamad.sawan@polymtl.ca; 2 Department of Electrical and Computer Engineering, McGill University, Montréal, Quebec, Canada H3A 2A7; E-mail: vamsy.chodavarapu@mcgill.ca; 3 Department of Chemical Engineering, École Polytechnique de Montréal, Montréal, Quebec, Canada H3S 3A7; E-mail: oumarou.savadogo@polymtl.ca

**Keywords:** Strain sensor, urinary bladder volume, polypyrrole, impedance spectroscopy, elastic properties, gauge factor

## Abstract

This paper describes a new technique to measure urine volume in patients with urinary bladder dysfunction. Polypyrrole – an electronically conducting polymer - is chemically deposited on a highly elastic fabric. This fabric, when placed around a phantom bladder, produced a reproducible change in electrical resistance on stretching. The resistance response to stretching is linear in 20%-40% strain variation. This change in resistance is influenced by chemical fabrication conditions. We also demonstrate the dynamic mechanical testing of the patterned polypyrrole on fabric in order to show the feasibility of passive interrogation of the strain sensor for biomedical sensing applications.

## 1. Introduction

Urinary bladder dysfunction continues to afflict millions worldwide. Often the result of devastating medical conditions is such as spinal cord injury, stroke and Parkinson's disease. Urinary bladder dysfunction not only leads to loss of voluntary control over the bladder muscles, but also cuts off sensorial feedback to the central nervous system [1]. This leaves patients incapable of sensing bladder fullness and gauging the right moment to trigger bladder voiding. Neural stimulation has been used to restore voluntary control of the bladder [2, 3]. Of the various techniques, stimulation of the sacral root at the base of the spine is considered the most efficient technique to produce micturition and has been prevalent in clinical practice over the last two decades, primarily for persons with spinal cord injury.

Direct sacral nerve stimulation has proved to be clinically viable through the work of several researchers. Among such devices is a dual implantable stimulator implemented by our research group [4]. This device is designed to permanently stimulate the pelvic floor in order to reduce (or eliminate) the bladder overactivity and to maintain the bladder function. The external controller of this system offers the option of choosing between two types of stimulation (permanent or selective) and customizing the stimuli parameters and for carrying out the stimulation. This neurostimulator is capable of producing selective stimulation and continuous low amplitude with a low frequency current for neuro-modulation of the bladder function and inhibiting detrusor overactivity. However, programmable neural stimulation is only one half of the solution in the rehabilitation of dysfunctional bladders. In particular, people afflicted with spinal cord injury are also deprived of sensorial feedback that could alert them to the fullness of the bladder and direct them to micturate when required. A truly functional urinary stimulator would have to possess the ability to trigger emptying of the bladder in response to maximal bladder volume.

**Figure 1.**
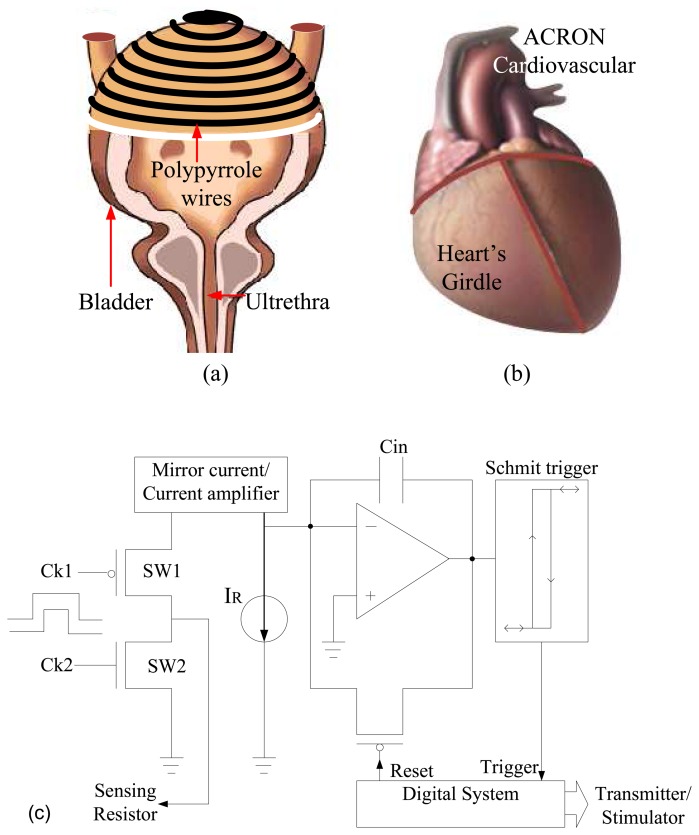
The strain sensor method: (a) illustration of bladder covered by stock with strip lines of PPY and (b) illustration of similar stock commercialized for heart failure problems, (c) an implantable circuit for reading out the sensing parameter.

Traditionally, catheterization has been a commonly used method for bladder volume assessment, but it is highly invasive and has the risk of infecting the urinary bladder. The advent of miniaturized devices and microelectronics has led to the development of a range of both non-invasive and implantable devices to measure the bladder capacity. In this paper, we propose a new method for bladder volume assessment using a conductive polymer as the sensing device. The conducting polymer – polypyrrole (PPY) – is coated on a flexible fabric and inserted over the upper portion of the bladder as shown in Figure 1a. This concept is similar to ACORN – a simple stocking placed over a weak heart to support and restore normal contractility (see Figure 1b). An implantable interface circuit is required to readout the changes in resistance. A high precision resistance measurement technique is a conventional Wheatsone bridge or its modified version [5]. However, due to required calibration procedure with off-chip resistors using Wheatstone method, a resistor-less technique such as the one shown in Figure 1c is more suitable [6]. In this technique the sensing current proportional to sensing resistor is extracted through the clocking system (Sw1, Sw2) which is amplified in the current mirror block and then integrated using a capacitor (Cin). The output of Schmitt Trigger block drives the digital counter which outputs a value proportional to input resistance value. A similar methodology has already been used to measure the capacitance for biosensing applications [7]. This digital value can either be transmitted to other implanted units or be used for stimulation purposes. In this paper, our focus is on the PPY-based strain sensor.

Conducting polymers such as polypyrrole have a long history of use in BioMEMS (*e.g.*, Polymer Actuators [8]) and biomedicine, most notably as neural interfaces and scaffolds for neural tissue growth. They have also been considered as candidates for “wearable” sensors and interfaces for biosensors and DNA chips. Polypyrrole is a positively-charged conjugated polymer. The attractiveness of this polymer for biomedical applications lies in its biocompatibility, ease of preparation, stability in air, and its ability to incorporate a wide variety of dopant ions.

Apart from its electron-conducting properties, this polymer also possesses the so-called intercalation property whereby entrapped counterions can be electrically released and incorporated when electrically activated. This is accompanied by a change in volume, thus making polypyrrole an attractive candidate for artificial muscles and drug delivery substrates. A simple method of polymerization of pyrolle on a flexible fabric is to first impregnate with an iron chloride solution. Thereafter, the fabric is dip-coated in a solution of pyrrole in acetonitrile and allowed to polymerize till a black film appears over the fabric. Alternatively, the polymerization can be carried out in the vapour phase. In this case, the fabric impregnated with iron chloride is suspended in a hermetically sealed jar containing the pyrrole solution. The jar is gently heated to 60^o^C and placed in a thermostat overnight. This technique allows formation of a thinner and more uniform layer of conducting polymer.

This paper is organised as follows: in Section II, we provide a brief review of other related works. In Section III, the details of our proposed method are presented. In section III, we describe the principle of proposed measurement technique. The experimental issues are presented in Section IV and followed by a discussion in Section V. Finally, a conclusion of this work is put forward in Section VI.

## 2. Techniques to Measure Bladder Volume

### 2.1. Pressure Sensors

Results presented by Koldewin *et al.* [9] demonstrate the possibility of using conventional strain gauges to measure pressure among the various placements of strain sensors, sensors that were placed between the peritoneum and muscular layer gave the best results. However, sensors placed between the mucosal and muscular layers were eroded quickly through bladder movement. During demonstration of the technique, the authors showed the pressure elevation caused by the accumulation of urine to be low and not reliable while considering the artefacts caused by a patient's movements.A more recent paper by Korkmaz and Rogg [10] on bladder mechanics shows that the urinary bladder has the ability to keep pressure virtually constant through stress relaxation, thus casting doubt on the efficacy of pressure measurements to determine volume.

### 2.2. Ultrasound Measurements

Acoustic measurements are integral to clinical neurodynamics and are widely used in measuring bladder volume. The underlying principle of this technique is the non-linear wave distortion between water and tissues. Among the various devices proposed was a portable miniaturized ultrasonic monitor by Petrican and Sawan [11] which allowed for estimation of the urine volume with an accuracy of 75%. The principle of this ultrasound system involved the detection of a certain threshold volume corresponding to 80% of the maximum daily capacity of the bladder. While in-vivo studies demonstrated the reliability of this device to detect a certain threshold volume in eneuretic patients within a reasonable margin of error, this system has not yet been demonstrated to measure intermediate volumes.

More recently, Merck *et al.* [12] described a multilayer transducer to assess bladder volume. In their efforts to optimize bandwidth and sensitivity of the device, a multilayer element consisting of a layer of polyvinylidene fluoride (PVDF) was glued onto a PZT transducer. The PVDF and PZT played the role of the broadband receiver and transmitter, respectively. By mechanically moving the transducer along the walls of a phantom bladder, this device records volumes of 133 ml and 500 ml. However, from this paper, it is less clear whether this device could measure a range of bladder volumes between these two thresholds

### 2.3. Bioelectric Impedance Measurement

A handful of research papers point to the measurement of bladder volume through bioelectric impedance both from inside and outside the body. In the case of implantable devices, this technique involves injecting an excitation current into the bladder and measuring the voltage induced between several electrodes fixed on the bladder wall. However, this approach has been shown to irritate the bladder muscle as it requires the placement of several electrodes on the detrusor muscle [13]. Also, the excitation current and direct placement of electrodes on the bladder might inadvertently lead to bladder muscle stimulation.

### 2.4. Electroneurogram Measurements

The monitoring of sensory nerve signals to control assistive devices was suggested by Stein *et al.* [10]. Since then, several researchers have reported peripheral sensory nerve signals recording from different applications [12-13]. The work of Stein *et al.* [14] showed that it is possible to record the electroneurogram (ENG) from cuff electrodes placed around the sacral roots which innervate the bladder during bladder filling, thus causing passive bladder distensions and reflex bladder contractions over a long period. The bladder wall tension depends on the detrusor pressure and the bladder volume. In rats and cats, it was observed that bladder afferent nerves increase their firing frequency during passive bladder distensions and during active bladder contractions. Recently, Intraoperative ENG monitoring of bladder fullness has been reported in humans using cuff electrodes. However, these observations have not yet resulted in a working device that would automatically reveal bladder volumes based on ENG recordings. Such a device would require complex signal extraction, processing and pattern recognition.

Based on the above discussion, it is clear that there exists a range of techniques to measure bladder volume, each with its advantages and drawbacks. It is also evident that none of these techniques factors in the elasticity of the bladder wall. Bladder walls are highly viscoelastic structures producing strain ratios of up to 70%, thus correlating well with the volume of the bladder. As a simple and sturdy method to measure volume change by exploiting the inherent viscoelasticity of the bladder, we have designed a volume sensor based on an electroconductive and elastic pouch that could be easily slipped around the urinary bladder, as shown in Figure 1a. The next section examines the underlying theoretical considerations that lead to such a design.

## 3. Mathematical Methodology of Proposed Strain Sensors

### A. Strain Sensor Basics

**Figure 2.**
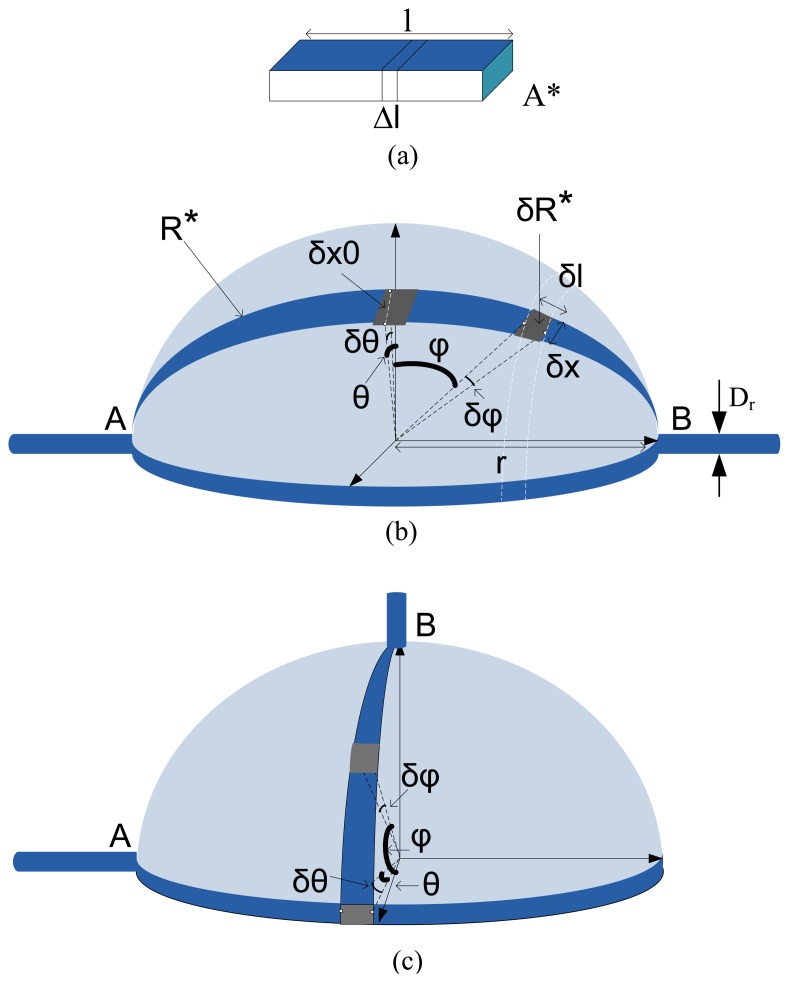
Illustration of different models for Strain Sensor: (a) simple strip-line, (b) and (c) spherical shape sensor with two different probing places (For simplicity, the bladder is assumed to be perfectly spherical).

Strain sensors are designed to convert a mechanical deformation to an electronic signal. The strain experienced by the sensor generally manifests itself as a change in capacitance, inductance or resistance, however, the dominant factor is resistive for polypyrrole coating layer exposed to humid environment around the bladder in the body. Let us assume a wire which is stretched within its elastic limit by a small amount, Δ1, such that its new length becomes 1+Δ1, as shown in Figure 2a. Thus, given that the electrical resistance is related to the length of the sensor, then the resistance of the stretched wire is given by

(1) $${\text{R}}_{\text{stretched}}=\rho \frac{1+\Delta 1}{{\text{A}}_{\text{stretched}}}$$

where ρ and A_stretched_ are the resistively constant and cross section area of resistor R_stretched_. The increase in the resistance of the stretched wire ΔR is

(2) $$\Delta R=R_{\text{stretched}}-\rho \frac{l}{A^{*}}$$

The fractional change in resistance divided by the fractional change in length is called the gauge factor G and is an indication of the sensitivity of the strain sensor.

(3) $$\text{G}=\frac{\Delta \text{R}/\text{R}}{\Delta 1/1}$$

Now, let us assume a half spherical shape strain sensor (see Figure 2b) and calculate the total resistance standing between node A and B (R_T_) as a function of volume change. For this, by dividing this three dimensional resistance to tiny and differential resistances (δR_θ_,φ) the total resistance can be calculated through serial or parallel configurations of these resistances. Figures 2a and 2b show two different models. As seen in these figures, the difference only is in the position of nodes A and B. In fact, as seen in Figure 2b, R* is the equivalent resistance of a segment of sphere ( all segments are evidently as the same).

In other words, the total resistance R_T_ is equal to the parallel of all R* values as shown in equation (4).

(4) $${\text{R}}_{\text{T}}=\frac{1}{\int_{\theta =0}^{\theta =\pi }\frac{1}{{\text{R}}^{*}}}$$

R* can be divided into small elements of δR* volume which only depends on φ (for 0<θ<π) or δR*=δRφ. Here, R* can also calculated as the series of δRφ. δRφ can be obtained from equation (5).

(5) $$\delta R_{\varphi }=\rho \frac{\delta l}{h.\delta x}$$

where, δl, δx and h are the differential length and width, and the thickness of differential segment as shown in Figure 2b. The minimum value of δRφ is obtained where δx=δx_0_ for φ=0. Also, δx for different values of φ are expressed by equation. 6 (see Appendix A).

(6) $$\delta x=\delta x_{0}\cdot \sin(90-\varphi )$$

Therefore, by assuming r as the radius of spherical shape resistor, sin(δφ)≈δφ , sin (δ θ)≈δθ, δl and δx_0_ are expressed by equations (7)-(8).

(7) $$\delta x_{0}=\delta \theta \cdot r$$

(8) δl=δφ⋅r

Therefore,

(9) $$\delta R_{\varphi }=\rho \frac{r\cdot \delta \varphi }{h\cdot r\cdot \delta \theta \cdot \sin(90-\varphi )}\Rightarrow \frac{\rho }{h}\cdot \frac{\delta \varphi }{\sin(90-\varphi )}=\delta R_{\varphi }\cdot \delta \theta$$

It is worth to mention that the connection of wires to strain sensor at nodes A and B are not only two dimensionless points. On other words, the cylindrical shape conductors with a diameter equal to D_r_ > 0 result in -π/2+φ_0_<φ<π/2-φ_0_ (see Figure 2b). By applying the integration operator on both sides of equation (9):

(10) $$\frac{\rho }{\text{h}}\cdot \int_{\varphi =\frac{\pi }{2}{+\varphi }_{0}}^{\varphi =\frac{\pi }{2}{-\varphi }_{0}}\frac{\delta \varphi }{\sin(90-\varphi )}=\delta \theta \int {\delta \text{R}}_{\varphi }$$

Therefore,

(11) $$\frac{2\rho }{\text{h}}\int_{\varphi =0}^{\varphi =\frac{\pi }{2}\varphi_{0}}\frac{\delta \varphi }{\cos(\varphi )}=\frac{2\rho }{\text{h}}\cdot \xi (\varphi_{0})=\text{R}*\cdot \delta \theta .$$

where φ_0_ # 0 and ξ(φ_0_) is obtained from equation (12).

(12) $$\begin{array}{l} \xi \left(\varphi_{0}\right)=\left[\varphi \cos^{-1}\varphi -\sqrt{1-\varphi^{2}}\right]|\begin{array}{l} \varphi =\frac{\pi }{2}-\varphi_{0}\\ \varphi =0 \end{array}\\ =1+\frac{\frac{\pi }{2}-\varphi_{0}}{\cos(\frac{\pi }{2}-\varphi_{0})}-\sqrt{1-{\left(\frac{\pi }{2}-\varphi_{0}\right)}^{2}} \end{array}$$

Therefore ξ(φ_0_) is valid for π/2>φ_0_>π/2-1, and it reflects a constant value for any φ_0_ in this range. Also, by substituting equation (12) in equation (11), R* can be obtained as a function of φ_0_ and consequently the total resistance of strain sensor is obtained from equation 4 as follows.

(13) $${\text{R}}_{\text{T}}=\frac{2\rho }{\pi \cdot \text{h}}\cdot \xi (\varphi_{0})$$

**Figure 3.**
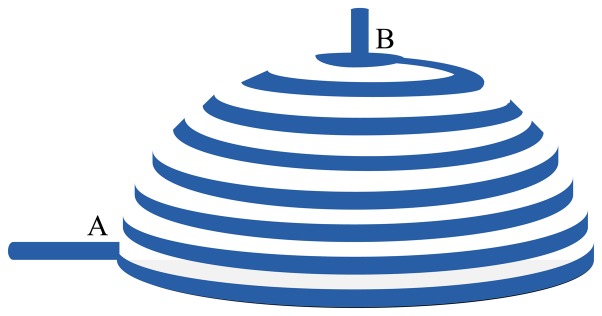
Illustration of different models for Strain Sensor with strip-line model.

Through these calculations, it is obvious that the total measured resistance cannot be a function of radius (or the volume) of spherical shape sensor. The same calculation can be performed on the model shown in Figure 2c that results in a similar conclusion. In fact, these calculations show the importance of the patterning of PPY on fabric for this application as shown in Figure 3.

On other words, the expansion in both width and length of each finite element do not allow a change in the summation of these elements. Based on above discussion, the only solution is to form the long and narrow strips of PPY on fabric so that the expansion of width can be ignored in comparing with length expansion (G=1). The patterning of PPY on fabric plays a critical role on the functionality of this strain sensor.

### B. Biomechanics of the Urinary Bladder

Like most soft tissues in the body, the urinary bladder wall is non-linear, viscoelastic and anisotropic. The collagen fibres are kinked and coiled when the bladder is relaxed and begin to stretch during filling. Correspondingly, the collagen fibres allow for high strain, which means that the urinary bladder can cater for a volume of up to 11 times its resting volume. While extensive urodynamic studies involving pressure changes during filling and voiding have been widely reported in the literature, less attention has been paid to the stress-strain relationships of the urinary bladder. To fill this void, Korkmaz and Rogg [10] used previous data in urodynamics to derive stress-strain relationships of the urinary bladder under various conditions. In all the results reported, the non-linear character of soft biological tissues manifests itself. During filling, the stress first increases slightly, and then rises extremely towards the end of the filling process. This behaviour of the urinary bladder is caused by the stiff collagen fibres which are increasingly stretched as the strain is increased. Due to the higher pressure difference during filling the stress is always larger for the first case than for the second case. Based on the above conditions, we set forth the following design requirements for the proposed strain sensor:

### C. Minimum Design Requirements for Bladder Volume Sensor

1. A non-linear viscoelastic fabric with a Young's modulus equal or greater than that of bladder tissue (as seen in result section)
2. High gauge factor and low threshold volumes of detection (G≥1)
3. Biocompatible and non-cytotoxic. ( PPY is biocompatible)
4. Easily implanted surgically with minimal sutures (Based on explanation in Fabrication section).

The ensuing sections detail the fabrication of our sensor that meets the above requirements, as well as its mechanical and electrical characterization and in-vitro results on phantom bladders.

## 4. Fabrication and Characterization of Sensor

In this section, the experimental procedures and results are described and demonstrated in the support of discussed issues throughout the paper.

### 4.1. Polypyrrole deposition

**Figure 4.**
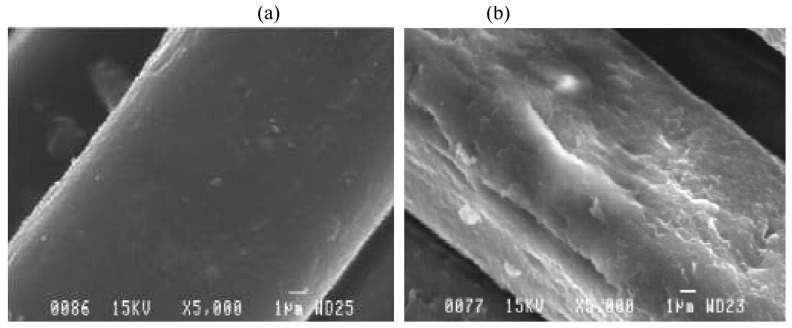
SEM images of (a) plain nylon fabric vs. same, but (b) polypyrrole-coated.

Two methods of fabrication were carried out. In the first method, a semi-circular pouch was cut out of commercially-available nylon hosiery. The fabric was soaked overnight in ethanol to remove residual dyes and other impurities. The dried fabric was then first soaked in a solution of 0.1 M lithium percholarate which serves as oxidising agent and a counter-ion. The fabric was then exposed to a solution of pyrrole monomer in deionised water and left for an hour to polymerize and permeate the fabric. In a second method, the polypyrrole was deposited through vapour phase polymerization: The polymer fabric (strips and pouches) was first soaked in a solution of ferric chloride and air-dried to allow for impregnation. Thereafter, a solution of pyrrole was placed in a closer vessel to which this fabric was suspended and placed in a thermostat at 60^o^C and left overnight to allow the pyrrole vapour to permeate the fabric and react with the impregnated oxidizing agents to form polypyrrole. For this paper, we use vapour-phase polymerization as it produced a more uniformly coated polypyrrole fabric that remains stable over many stress- strain cycles (Figure 4). The patterning of ppy on fabric can be easily preformed through digital dispensing system or direct-write technique [15]. For this purpose, the pre-prepared ppy is extruded from a micronozzle in the desired trajectory using a robot (R&J Inc.) to carry the syringe and nozzle.

### 4.2. Tensile Properties of Strain Sensor

**Figure 5.**
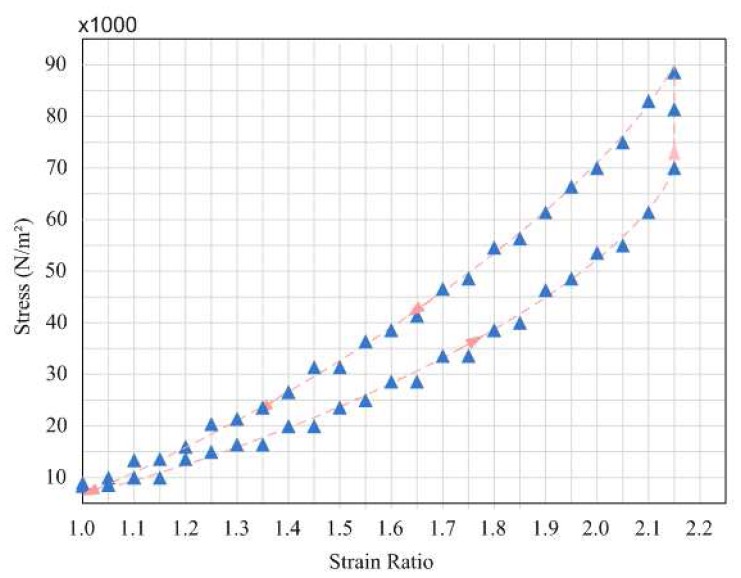
Stress vs. Strain in Polypyrrole- Coated Nylon Fabric.

The uniaxial strain properties of the fabric were tested as described below. A 10x2 cm strip of Ppy-coated fabric was cut with the ends rolled against two nails and suspended into the sample holder. A Bionix X Mechanical Analyzer was used to measure the strain properties of the polymer as a function of stress. The fabric was analyzed in the strain mode at a strain rate of 100 mm/min. The resulting stress-strain curve of the fabric is shown in Figure 5. From this Figure, we notice the same non-linear curve characteristic of the bladder walls as in the case of the fabric. The calculated value of the Young's modulus is 25, comparable to that of the bladder wall.

### 4.3. In-Vitro Testing of Strain Sensor on Bladder Volume

**Figure 6.**
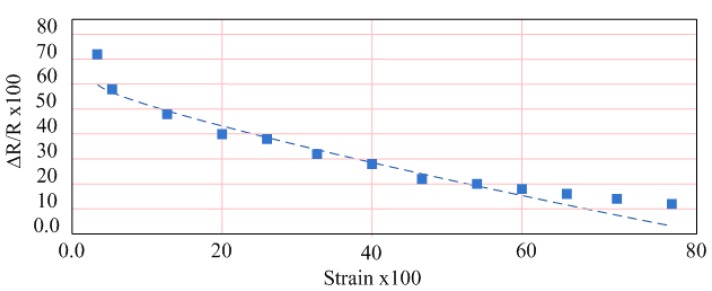
Resistance vs. Strain in Polypyrrole-Coated Strain Sensor.

To measure the electrical properties of strain sensors a rubber balloon was transfixed on to a stand leaving the aperture free for water intake. Strips of polypyrrole-coated fabric were glued onto an insulating layer of the same fabric, which was fixed onto the balloon wall. The ends of the fabric were attached to a Fluke 189 Multimeter for recording. Measured volumes of water were gradually added to the balloon and a change in resistance of the fabric was noted as the balloon walls stiffened and expanded. The measurement approximately shows a linear resistance response was produced in the 40-100 ml range (see Figure 6). Since the urinary bladder walls are highly elastic structures with an elastic modulus, it is appropriate that a strain sensor to measure bladder volume be at least as elastic as was demonstrated in the case of the PPY-coated nylon fabric.

Specifically, the strain sensor produced a linear response and sensitivity in at 100-300 ml which corresponds to the range at which the normal micturition reflex takes place. Different fabrication techniques and chemical modifications of the sensor were carried out to optimise the strain sensor for stability, sensitivity, conductivity and reversibility. The thin bladder strips produce an equivalent response regardless of their location for as long as their fibres are aligned vertically along the wall. The use of a conductive pouch resolves the conundrum of placing the sensor at the right place. The pouch design is such that it fits snugly over the bladder with minimal or no surgical sutures.

## 5. Discussion and Future Work

In this section, the practical considerations regarding the sensor and further in-vivo issues are discussed.

### 5.1. In-vivo consecrations

The urinary bladder is a highly sophisticated organ with an intricate control and feedback mechanism through an extensive network of efferent and afferent pathways modulating the movement of the bladder. While urinary implants have effectively dealt with the stimulation aspect, little attention has been drawn to devices that can substitute sensory feedback and alert the patient to the appropriate time to micturate. Measurements of volume are critical to restore voluntary control of the bladder. The method proposed in this paper exploits the unique visoelastic properties of the bladder walls. The urinary bladder is a highly elastic structure able to accommodate up to eleven times its rest volume. So, while the pressure of the bladder walls changes very little, the strain response of the bladder walls would be a reliable indicator of urine volume. This is the underlying reason for designing a sensor that would be both elastic and responsive to volume change.

As already mentioned, the Interrogation or reading out of the sensor can be carried out in two different ways. In the first instance, an implantable circuit can provide continuous resistance outputs for a given input voltage. This resistance reading can then be transmitted wirelessly to a wearable display positioned just outside the body. Alternatively, if the resistance of the strain sensor can be substantially decreased through chemical modification, the resistance of the strain sensor could then be interrogated passively through a resonance circuit located outside the body. This would obviate the need for implanted circuits and batteries.

### 5.2. Strain sensor practical issues

Conducting polymers widely used as sensors, actuators and super-capacitors are inherently rigid. Only recently, with the advent of so-called wearable sensors and electronic textiles has the notion of conductive fabric come to light. While PPY-coated fabric been studied for wearable strain gauges, the implantable applications have yet to be studied. This work represents, to the best of our knowledge, an innovation in implantable devices for this particular clinical need. The mechanical properties in Figure 5 show that a non-linear profile stress-strain with modulus of elasticity up to comparable to that of the bladder wall. The fabrication technique is key component to optimizing the properties of the strain sensor. Strain sensors rely on conductivity, sensitivity, stability and reproducibility. We used a standard solution phase polymerization and vapour phase polymerization. The differential behaviour of these two is shown in Figure 5. The images of the fabric (see Figure 4) show a clear impregnation within individual fibres, thus attesting to the stability of the fibre. Yet another advantage of this technique is the inherent nature of a dysfunctional bladder. Unlike healthy bladders that become nearly spherical when full, dysfunctional bladders are irregularly-shaped. A strain sensor that accommodates the shape of the bladder, such as the polypyrrole sensor, offers a clear advantage over traditional rigid sensors.

As would be expected, the solution-phase polymerization produced a thick and non-uniform layer as compared to that obtained through vapour-phase polymerization. While still wet, both fabric strain showed resistance values of 6-10 kΩ. However, when dry, the resistance of the sample increased 5-fold and after a few days did not produce any change in resistance on stretching. The explanation could be as follows: water acts as a percolating medium between the polypyrrole-coated fabrics and enhances the conductivity between them. When dried, the fabric might create disconnected domains without any percolating medium between them. On the other hand, polypyrrole vapour has a higher permeability in the fabric and produces thinner and more stable and continuous films.

In designing the optimal shape of the strain sensor, we first used thin strip of 10 cm x 3 cm. However, the strain response remained the same as long as it was vertically placed and the fibre alignment along the walls of the phantom bladder. Thus, we propose a pouch configuration. There exists an opening on top of the bladder between the urethral tubes which would allow for easy slipping of the semi-circular pouch with minimal or no surgical sutures. As a future work, to develop a fully functioning system, we propose to fabricate an interface chip as shown in Figure 1c, followed by in-vivo testing of the integrated system.

## 6. Conclusions

This paper demonstrated the viability of using an electroconductive polymer fabric to measure the volume of a phantom bladder. The polypyrrole-coated nylon fabric is both electrically conductive and highly elastic, akin to bladder tissues. A review of previous techniques points to their limitations. Certain parameters are either insensitive to bladder volume, such as pressure, too invasive and subject to movement such as electrochemical impedance and requires mechanical displacement of an external acoustic transducer to obtain the range of intermediate volumes.

## Figures and Tables

**Figure 7.**
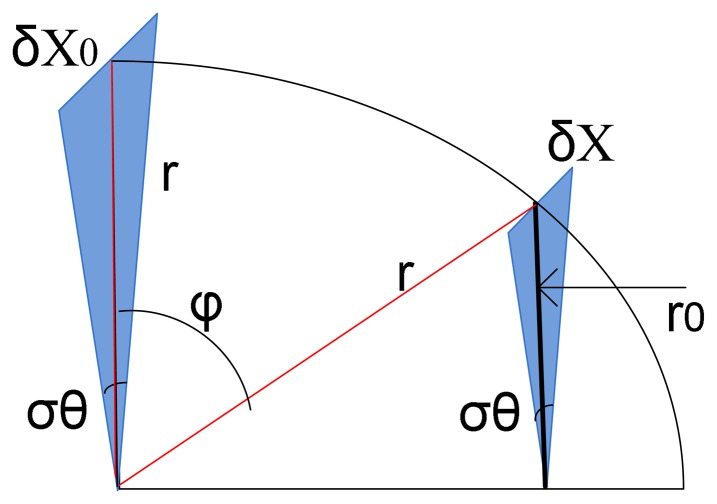
An Illustration for supporting the calculations.

## Appendix A

Based on Figure 7, the following equations can be derived.

$$\frac{\delta x}{r}=\frac{\delta x_{0}}{r_{0}}=\sin(\sigma \theta )$$

$$\frac{r_{0}}{r}=\sin(90-\varphi )$$

By combining both equations, the following equation can be expressed.

$${\delta \text{x}=\delta \text{x}}_{0}\cdot \sin(90-\varphi )$$
